# Improved WαSH Feature Matching Based on 2D-DWT for Stereo Remote Sensing Images

**DOI:** 10.3390/s18103494

**Published:** 2018-10-16

**Authors:** Mei Yu, Kazhong Deng, Huachao Yang, Changbiao Qin

**Affiliations:** 1 NASG Key Laboratory of Land and Environment and Disaster Monitoring, China University of Mining and Technology, Xuzhou 221116, China; tb15160011b2@cumt.edu.cn (M.Y.); 3868@cumt.edu.cn (H.Y.); 2 School of Environment Science and Spatial Informatics, China University of Mining and Technology, Xuzhou 221116, China; 3 Jiangsu Key Laboratory of Resources and Environmental Information Engineering, China University of Mining and Technology, Xuzhou 221116, China; 4 Wits Mining Institute, University of the Witwatersrand, Johannesburg 2050, South Africa; 1583615@students.wits.ac.za

**Keywords:** stereo remote sensing image, feature matching, 2D-DWT, WαSH, MSER, image deformation

## Abstract

Image matching is an outstanding issue because of the existing of geometric and radiometric distortion in stereo remote sensing images. Weighted α-shape (WαSH) local invariant features are tolerant to image rotation, scale change, affine deformation, illumination change, and blurring. However, since the number of WαSH features is small, it is difficult to get enough matches to estimate the satisfactory homography matrix or fundamental matrix. In addition, the WαSH detector is extremely sensitive to image noise because it is built on sampled edges. Considering the shortcomings of the WαSH detector, this paper improves the WαSH feature matching method based on the 2D discrete wavelet transform (2D-DWT). The method firstly performs 2D-DWT on the image, and then detects WαSH features on the transformed images. According to the methods of descriptor construction for WαSH features, three matching methods on the basis of wavelet transform WαSH features (WWF), improved wavelet transform WαSH features (IWWF), and layered IWWF (LIWWF) are distinguished with respect to the character of the sub-images. The experimental results on the dataset containing affine distortion, scale distortion, illumination change, and noise images, showed that the proposed methods acquired more matches and better stableness than WαSH. Experimentation on remote sensing images with less affine distortion and slight noise showed that the proposed methods obtained the correct matching rate greater than 90%. For images containing severe distortion, KAZE obtained a 35.71% correct matching rate, which is unacceptable for calculating the homography matrix, while IWWF achieved a 71.42% correct matching rate. IWWF was the only method that achieved the correct matching rate of no less than 50% for all four test stereo remote sensing image pairs and was the most stable compared to MSER, DWT-MSER, WαSH, DWT-WαSH, KAZE, WWF, and LIWWF.

## 1. Introduction

Image feature matching, which aims to acquire reliable homonymous features—matches between images—is one of the most basic and important processes of remote sensing image processing [1,2]. The accuracy of matching results directly affects the reliability of image registration [3] and fusion, automatic moving objects detection [4], and aerial triangulation and 3D reconstruction [5].

Feature matching includes three steps: feature detecting, feature describing, and similarity matching. At present, the most widely used feature matching algorithm is the Scale Invariant Feature Transform (SIFT) algorithm [6] which firstly uses the difference-of-Gaussian (DOG) operator to identify potential interest features, followed by features localization and orientation assignment, and then describes the features according to local image gradients, finally performing the similarity matching to acquire matches. The SIFT algorithm is adaptable to distortions, such as scale, rotation, translation, etc. Therefore, it is used in most matching processes of aerial or aerospace images. Yu et al. [1] employed the SIFT algorithm to create a set of matches and then removed outliers from the matches and the estimated the transformation relationship between unmanned aerial vehicle (UAV) images by using a maximum likelihood framework. It achieved the feature matching of UAV images under non-rigid transformation in precision agriculture. Chang et al. [7] combined the SIFT algorithm with scale constraints and geometric constraints to achieve highly-accurate registration of high-resolution satellite image. Xiang et al. [8] used a SIFT-liked matching algorithm to extract reliable matches for GF-3 SAR images and then provided a coarse-to-fine registration algorithm, giving a robust, efficient and precise performance of SAR image. Qi and Zhu [9] decomposed remote sensing images by discrete wavelet transform (DWT), and extracted the SIFT features on low-frequency sub-images, then used a coarse-to-fine strategy to improve the precision and efficiency of SIFT.

However, because of the effect of affine distortion as a result of the camera view change at the time of image acquisition, the matching performance of SIFT algorithm decreases sharply result in that reliable potential matches cannot be obtained [10,11]. In this case, affine-invariant features, such as Harris-Affine [12], maximally stable extremal region (MSER) [13], and edge-based region (EBR) [14,15] are required. Mikolajczyk et al. [16] compared six common affine-invariant feature detectors in which the results showed that MSER performs best in most cases and does well on images containing homogeneous regions with distinctive boundaries. Based on the MSER detector, Zhang et al. [17] proposed an automatic coarse-to-fine image registration strategy that achieves high accuracy for images containing affine distortion. Sedaghat and Ebadi [18] extracted the MSER and Harris-Affine features and selected the initial matches using the Euclidean distance between feature descriptors, followed by a consistency check process. Then oriented least square matching is developed to improve the positional accuracy of the affine-invariant features. Their affine-invariant image matching method was successfully applied for various synthetic and real close range and satellite images matching. Yu et al. [19] acquired the initial matches based on the MSER detector and calculated the affine transformations between the initial matches. A small number of high-accuracy matches were detected by combining the SIFT feature matching under the constraint of affine transformation and the neighborhood supporting principle. Then the propagation is performed based on the global affine transformation constraints to find more matches satisfying the accuracy registration of oblique images.

The weighted α-shape (WαSH) [20,21] algorithm has certain stability to the geometric and radiometric distortion of images and can detect invariant local features with high uniqueness. Compared with Hessian-Affine [12], MSER, medial feature detector (MFD) [22], edge foci (EF) [23], and KAZE algorithms [24], Varytimidis et al. [20] found that the WαSH feature detection algorithm performs among the best in all cases of image distortion through a comprehensive analysis of factors, such as repeatability, matching score, and number of matches.

**Figure 1:**
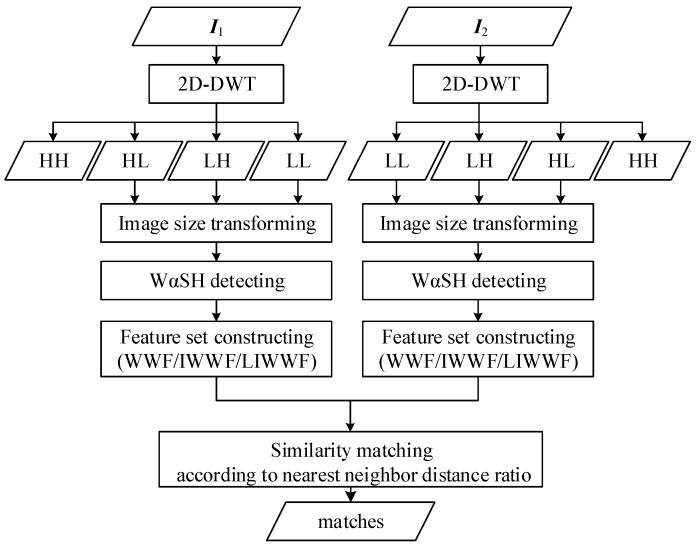
Procedure of improved WαSH feature matching based on 2D-DWT. ***I***_1_ and ***I***_2_ are the reference image and the image to be matched, respectively.

However, there also are some shortcomings of WαSH feature matching: (1) When there are large geometric distortions between images, including affine changes and scale changes, WαSH feature matching detects a few matches that contain a significant proportion of false matches and causes failure in calculating the transformation model between image pairs; (2) Since the WαSH features are extracted based on the image edge information, the repeatability and matching score will be greatly decreased if there is significant noise in images. In order to deal with the problems above, we improve the WαSH feature matching method based on 2D-DWT. This method firstly performs 2D-DWT decomposition on the original image and obtains four sub-images: LL, LH, HL, and HH. We discard the HH layer in which the noise in the original image is concentrated and transform the remaining sub-images to the original image size. WαSH detector is then performed on the transformed images. Depending on the descriptor construction methods for the WαSH features, three matching methods on the basis of wavelet transform WαSH feature (WWF), improved wavelet transform WαSH feature (IWWF), and layered IWWF (LIWWF), respectively, are distinguished. The matches are finally determined by using similarity matching. The procedure of improved WαSH feature matching based on 2D-DWT is illustrated in Figure 1.

## 2. 2D-DWT

DWT of an image transforms the image data from the spatial domain to frequency domain by using hierarchical decomposing functions [25]. A 2D decomposition is achieved by sequentially applying 1D-DWT along horizontal and vertical directions, respectively [26]. In a 1D decomposition, there are both low-(L) and high-pass (H) filters along each direction, which leads to one “L” and one “H” sub-band, respectively. The “L” sub-band corresponds to low-frequency components in the wavelet domain, while the “H” sub-band to high-frequency ones. After doing the first level 2D decomposition, there are four sub-bands in the wavelet domain, which are labeled as LL, LH, HL, and HH, respectively. 

The LL sub-band is generated by convolving the low-pass wavelet filter along horizontal and vertical directions on the image. It is an approximate representation of the original image. The LH sub-band is generated by convolving a low-pass wavelet filter in the horizontal direction and then convolving a high-pass wavelet filter in the vertical direction on the image. It represents a character in the vertical direction of the original image. The HL sub-band is generated by convolving a low-pass wavelet filter in the horizontal direction after a convolution of high-pass wavelet filter in the vertical direction on the image. It represents the horizontal characteristic of the original image. The HH sub-band is a wavelet coefficient generated by convolving high-pass wavelet filters in two directions on the image. It represents the diagonal edge characteristics of the image. Therefore, the LL sub-band contains most features of the original image, while the noise in the original image is concentrated in the HH sub-band.

## 3. WαSH Feature Detector

The WαSH detector exploits stable and distinctive dominant structural features in an image on the basis of image edge and α shape. It can be divided into four parts, namely edge detecting, edge sampling, WαSH constructing and feature extracting. The processes are as follows:

(1) Edge detecting: g is the normalized gradient image of the input image in [0,1]. The binary edge image e is acquired by applying the Canny detector on g.
(2) Edge sampling: With a fixed sampling interval s, e is sampled uniformly along edges to obtain a discrete set of edge points $P\subseteq R^{2}$. For each point p∈P, the weight w(p) is defined to be multiple of its gradient strength:
  (1) $$w(p)=g(p){\left(\frac{s}{2}\right)}^{2},$$
  where g(p)∈[0,1] is the value of g at p.
(3) WαSH construction: Regular triangulation of P is calculated. The line segments and triangles of triangulation are added into a collection K and are ordered by descending size, followed by the weighted α-shape constructing. For more details, please see [19].
(4) Feature extracting: the neighbors of each triangle $\sigma_{T}\in K$ are its three edges, while the neighbors of each line segments $\sigma_{E}\in K$ are the two adjacent triangles in the triangulation. Since the size of an edge is not larger than that of its two adjacent triangles, and α is decreasing, the intuition is that this edge can keep the two triangles disconnected until it is processed itself. Based on this neighborhood system, each connected weighted α complex is called a component. Considering that each element (line segment or triangle) of K in descending order of size is an independent component, the components are joined with their neighbors that have already been processed. Decreasing the value of α continuously, the strength of a component $k_{U}$ is calculated following Equation (2) when it is joined to another component through its boundary element:
  (2) $$s(k_{U})=\frac{a(k_{U})}{\rho_{T}},$$
  where $a(k_{U})$ is the total area of $k_{U}$ (the area of the line segment is 0); $\rho_{T}$ is the size of the boundary element. If strength is greater than a fixed threshold τ, the component is determined to be a WαSH feature which is consequently fitted to an ellipse. We assume the image coordinate of the ellipse center is (x,y), and the ellipse parameter is (a,b,c), then the ellipse equation is a(x−u)(x−u)+2b(x−u)(y−v)+c(y−v)(y−v)=1. The WαSH feature is denoted as $x={\left(x,y,a,b,c\right)}^{T}$ in this paper.

## 4. Algorithm

### 4.1. Feature Set Construction

**Figure 2:**
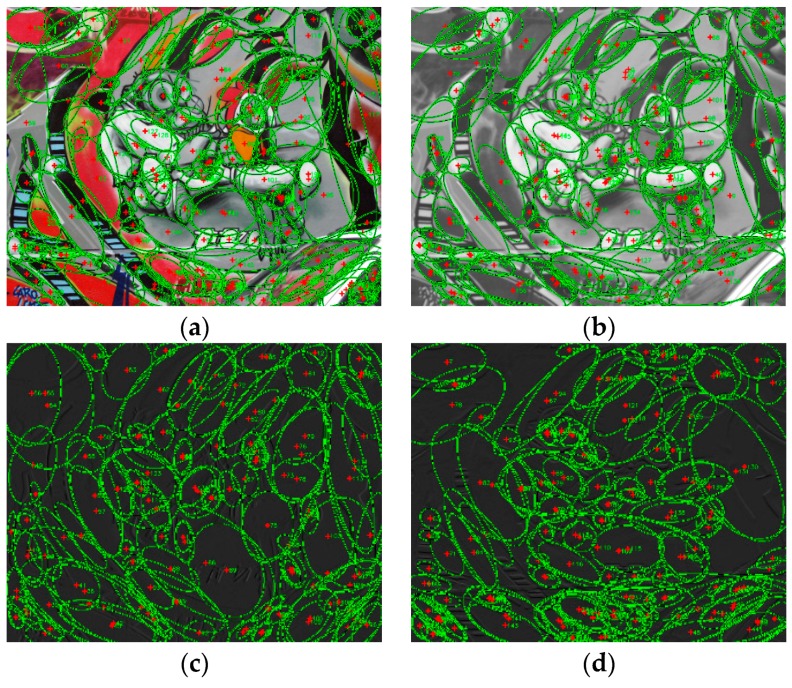
WαSH features on (**a**) original image, (**b**) LL layer, (**c**) LH layer, and (**d**) HL layer.

Wavelet transform (the Haar wavelet base is used in this paper) is firstly performed on the reference image and the image to be matched to obtain four sub-bands—LL, LH, HL, and HH. Since the sub-bands are two-dimensional, we consider them as four images of the LL, LH, HL, and HH layer. The LL layer image retains the original image content information, the LH layer, and the HL layer, respectively, maintain the vertical and horizontal information on the original image after wavelet transform. They are favorable for edge information and WαSH local features detecting. The noise in the original image is concentrated in the HH layer. Therefore, we perform WαSH detector on the images of LL layer, LH layer, and HL layer while discarding the HH layer. In order to avoid inconsistent coordinates, we transform the sizes of three-layer images to be equal to the original image. We perform the WαSH detection on the images of the LL layer, LH layer, and HL layer, and obtain the WαSH feature sets $S^{\mathrm{LL}}=\{x_{i}^{\mathrm{LL}}|i=0,1,\ldots ,n^{\mathrm{LL}}\}$, $S^{\mathrm{LH}}=\{x_{i}^{\mathrm{LH}}|i=0,1,\ldots ,n^{\mathrm{LH}}\}$, $S^{\mathrm{HL}}=\{x_{i}^{\mathrm{HL}}|i=0,1,\ldots ,n^{\mathrm{HL}}\}$, respectively, as displayed in Figure 2. 

According to the normalization theory in [16], the WαSH feature, an elliptical region, can be normalized into a circular one, on which the description of the feature is more robust to affine distortion.

A variety of feature describing operators were compared in [27], reaching the conclusion that SIFT-based description operators have better robustness than others. Therefore, we use the SIFT algorithm to calculate the descriptors for WαSH features.

Three feature sets—the wavelet transform WαSH feature (WWF) set, the improved wavelet transform WαSH feature (IWWF) set, and the layered IWWF (LIWWF) set are constructed based on the above-mentioned WαSH local invariant features detected on the images of LL layer, LH layer, and HL layer in this paper. As the features consist of coordinates on the image x and the description vectors d, we then denote the features using the representation of f=(x,d). In the following context, we use a superscript to distinguish different feature sets constructing method and a subscript to distinguish the original image for feature sets M containing descriptors.

#### 4.1.1. WWF Set Constructing

**Figure 3:**
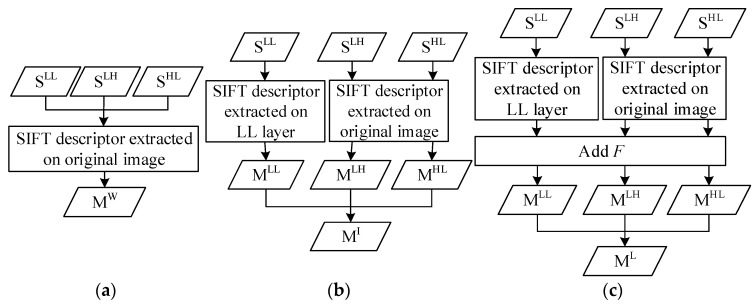
Feature set constructing process of (**a**) WWF, (**b**) IWWF, and (**c**) LIWWF.

Set $S^{\mathrm{LL}}$, $S^{\mathrm{LH}}$, and $S^{\mathrm{HL}}$ are combined into set $S^{W}=\{x_{i}^{W}|i=0,1,\ldots ,n^{W}\}$, where $n^{W}=n^{\mathrm{LL}}+n^{\mathrm{LH}}+n^{\mathrm{HL}}$ is the number of features in WWF. The description vector of feature $x_{i}^{W}$ is calculated by using SIFT algorithm on original image. Then the set of WWF is acquired and denoted as $M^{W}=\{f_{i}^{W}|i=0,1,\ldots ,n^{W}\}$, where $f_{i}^{W}=(x_{i}^{W},d_{i}^{W})$. The process is demonstrated in Figure 3a.

#### 4.1.2. IWWF Set Constructing

For $x_{i}^{\mathrm{LL}}$, the features on the image of LL layer, the description vector is calculated on the image of LL layer, while the description vectors of $x_{i}^{\mathrm{LH}}$ and $x_{i}^{\mathrm{HL}}$ are calculated on the original image due to the lack of image information on LH and HL layers. We acquired three sets $M^{\mathrm{LL}}=\{f_{i}^{\mathrm{LL}}|i=0,1,\ldots ,n^{\mathrm{LL}}\}$, $M^{\mathrm{LH}}=\{f_{i}^{\mathrm{LH}}|i=0,1,\ldots ,n^{\mathrm{LH}}\}$ and $M^{\mathrm{HL}}=\{f_{i}^{\mathrm{HL}}|i=0,1,\ldots ,n^{\mathrm{HL}}\}$, where $f_{i}^{\mathrm{LL}}=(x_{i}^{\mathrm{LL}},d_{i}^{\mathrm{LL}})$, $f_{i}^{\mathrm{LH}}=(x_{i}^{\mathrm{LH}},d_{i}^{\mathrm{LH}})$ and $f_{i}^{\mathrm{HL}}=(x_{i}^{\mathrm{HL}},d_{i}^{\mathrm{HL}})$ respectively. Merging sets $M^{\mathrm{LL}}$, $M^{\mathrm{LH}}$, and $M^{\mathrm{HL}}$, the set of IWWF is obtained and denoted as $M^{I}=\{f_{i}^{I}|i=0,1,\ldots ,n^{I}\}$, where $n^{I}=n^{\mathrm{LL}}+n^{\mathrm{LH}}+n^{\mathrm{HL}}$ is the number of features in IWWF. The process is demonstrated in Figure 3b.

#### 4.1.3. LIWWF Set Constructing

Similar to IWWF set constructing method, the description vectors of features in $S^{\mathrm{LL}}$ are calculated on the image of LL layer, the descriptor vectors of features in $S^{\mathrm{LH}}$ and $S^{\mathrm{HL}}$ are calculated on the original image. The difference with IWWF set constructing method is that a variable F is introduced to distinguish the layer to which the features belong in LIWWF. Therefore, we obtain sets $M^{\mathrm{LL}}=\{(f_{i}^{\mathrm{LL}},F)|i=0,1,\ldots ,n^{\mathrm{LL}},F=1\}$, $M^{\mathrm{LH}}=\{(f_{i}^{\mathrm{LH}},F)|i=0,1,\ldots ,n^{\mathrm{LH}},F=2\}$, and $M^{\mathrm{HL}}=\{(f_{i}^{\mathrm{HL}},F)|i=0,1,\ldots ,n^{\mathrm{HL}},F=3\}$, respectively. Merging sets $M^{\mathrm{LL}}$, $M^{\mathrm{LH}}$, and $M^{\mathrm{HL}}$, the set of LIWWF is obtained and denoted as $M^{L}=\{(f_{i}^{L},F)|i=0,1,\ldots ,n^{L},F=1,2,3\}$, where $n^{L}=n^{\mathrm{LL}}+n^{\mathrm{LH}}+n^{\mathrm{HL}}$ is the number of features in LIWWF. The process is demonstrated in Figure 3c.

### 4.2. Similarly Matching

The WWF sets $M_{1}^{W}$ and $M_{2}^{W}$ are constructed separately for the reference image $I_{1}$ and the image to be matched $I_{2}$, and the number of features are $n_{1}^{W}$ and $n_{2}^{W}$. For each element in $M_{1}^{W}$, the Euclidean distance between the feature description vector $d_{i}$ and all of the feature description vector $d_{j}$ in $M_{2}^{W}$ are calculated. If the nearest distance to nearest neighbor distance ratio (NNDR) between the $d_{i}$ and $d_{j}$ is less than 0.8, the corresponding features with the minimum distance in $M_{1}^{W}$ and $M_{2}^{W}$ are determined as a match. For sets IWWF of $I_{1}$ and $I_{2}$, the matches are determined in the same process with that in WWF. For sets LIWWF of $I_{1}$ and $I_{2}$, we first compare the values of F. If the values of F are equal, the Euclidean distance is calculated of the two description vectors in $M_{1}^{W}$ and $M_{2}^{W}$. If not, we set the distance to infinity and skip the distance calculation. Finally, the corresponding features are determined under the same criteria as that of WWF.

## 5. Experiments and Analysis

### 5.1. Performance Compared to WαSH for Different Distortion 

**Figure 4:**
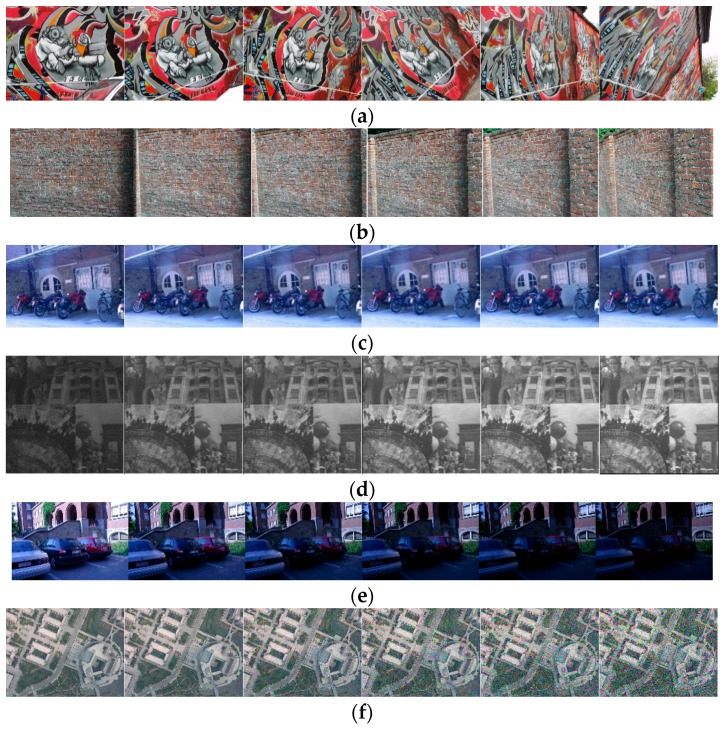
Test image sequences with affine distortion, scale distortion, illumination change and noise change in images. (**a**) graffti: the image affine distortion increasing from left to right; (**b**) wall: the image affine distortion increasing from left to right; (**c**) bikes: the image scale distortion increasing from left to right; (**d**) mosaic: the image illumination increasing from left to right; (**e**) Leuven: the image illumination decreasing from left to right; (**f**) library: the image noise increasing from left to right.

Six representative image sequences in the Krystian Mikolajczyk’s personal homepage [28] with affine distortion, scale distortion, and illumination change, and a group of UAV image pairs with different levels of noise are employed to test the performance of WαSH, WWF, IWWF, and LIWWF. The test images are shown in Figure 4 in which each sequence contains six images.

**Figure 5:**
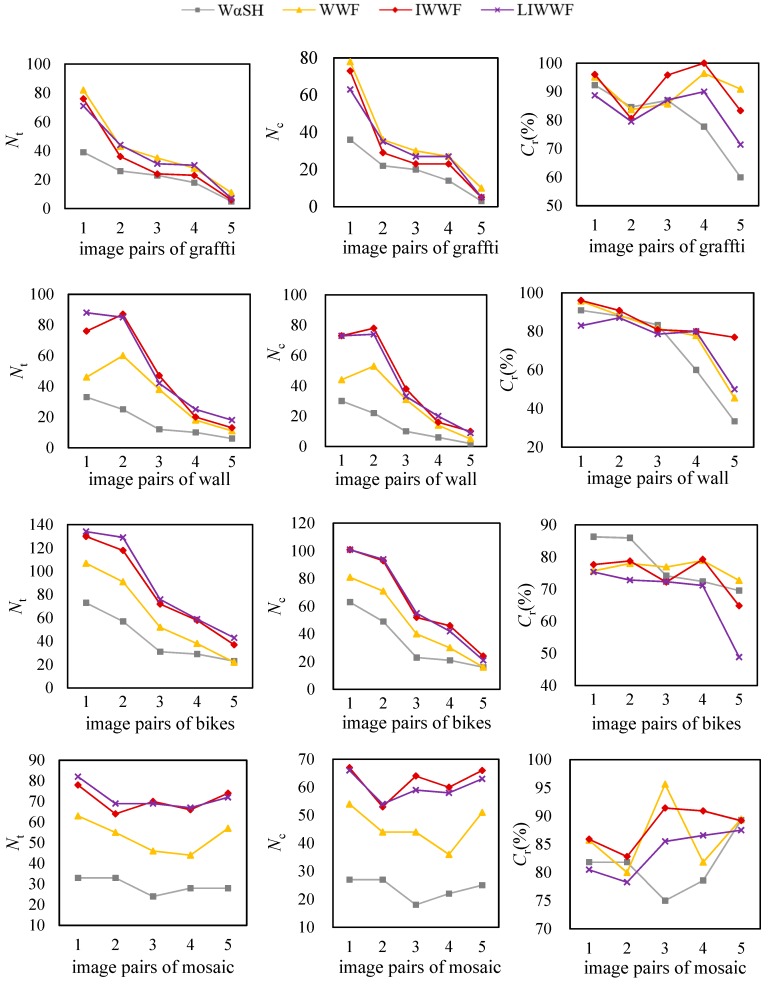

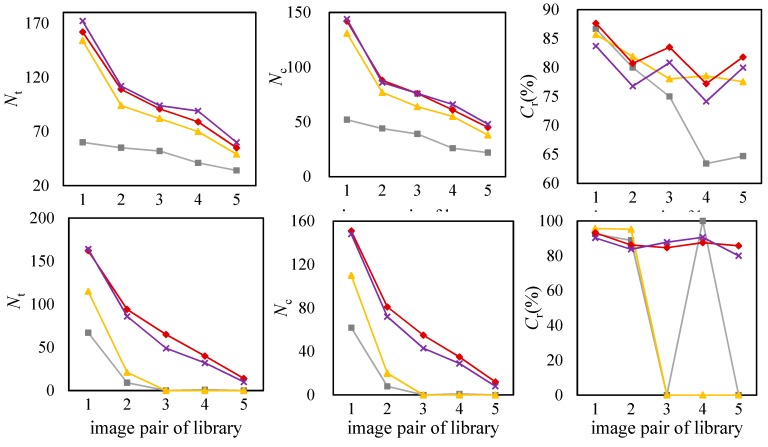
Results of different matching methods for the test data. *N*_t_: number of total matches; *N*_c_: the number of correct matches; and *C*_r_: correct matching rate (%).

The number of total matches (*N*_t_), the number of correct matches (*N*_c_) and correct matching rate (*C*_r_) are employed to evaluate the performances of the four methods. The matching results are shown in Figure 5, of which the 1, 2, 3, 4, 5 of the horizontal axis are different image pairs representing image 2 to 1, 3 to 1, 4 to 1, 5 to 1, and 6 to 1, respectively. *C*_r_ is calculated as the ratio percentage of *N*_c_ to *N*_t_. According to [16], a pair of matches was determined to be the correct one when the overlap error is less than 50%. 

According to the above experimental results, the analysis is as follow:

From Figure 5, we can find that the similar orders and changes occurred in the plots of the number of total matches and correct matches. WWF, IWWF, and LIWWF offered higher *N*_t_ and *C*_r_ than WαSH for most image pairs. That is because the improved methods use features on images of LL layer, LH layer, and HL layer which contain obvious image structure characteristics. Combining the features on all of three sub-images, WWF, IWWF, and LIWWF achieved more matches than the original WαSH method.

**Figure 6:**
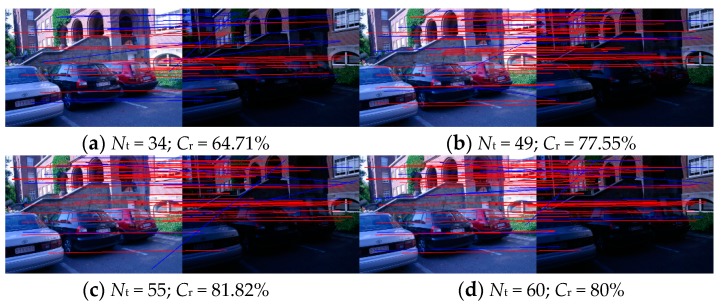
Results of (**a**) WαSH, (**b**) WWF, (**c**) IWWF, and (**d**) LIWWF for the 5th image pair of Leuven. The red lines indicate correct matches while the blue lines are incorrect matches.

Take the 5th image pair of Leuven in Figure 5 for example. The results of WαSH, WWF, IWWF, and LIWWF for this image pair are shown in Figure 6, and the repeatability and correct matching rate are displayed in Table 1. The repeatability measures how large proportion of the detected features is the corresponding scene region. The correct matching rate assesses the distinctiveness of the detected features.

Table 1 shows the WαSH features on LL and LH layer had a relative high repeatability. The reason is that the image of LL and LH layer contains obvious image structure characteristics. In addition, the matching accuracy of features described on LL layer was higher than the matching accuracy of features described on the original image. That means the image context of the LL layer is more distinctive than the image context of the original image in this case. Therefore, the *C*_r_ scores of IWWF and LIWWF are higher than the *C*_r_ scores of WαSH and WWF. 

### 5.2. Application for Stereo Remote Sensing Images Matching

**Figure 7:**
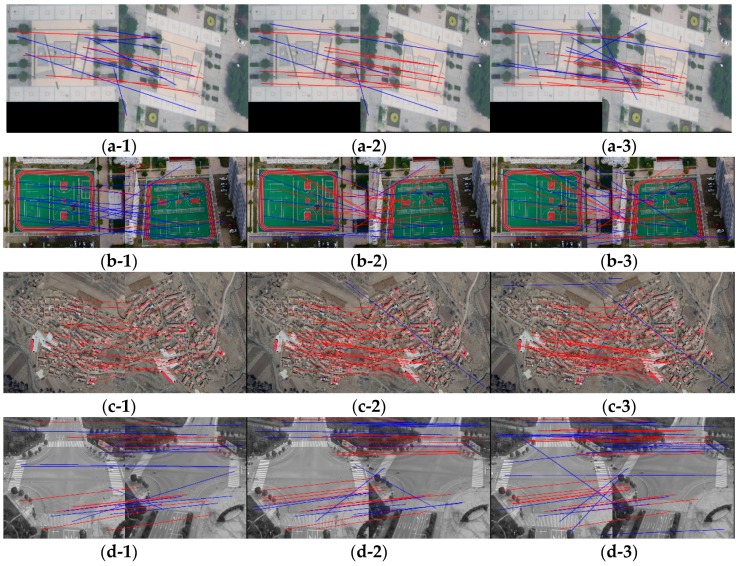
Results of proposed methods for remote sensing images obtained by UAV. The red lines indicate correct matches while the blue lines are incorrect matches. (**a-1**), (**b-1**), (**c-1**) and (**d-1**) display result of WWF; (**a-2**), (**b-2**), (**c-2**) and (**d-2**) display result of IWWF; (**a-3**), (**b-3**), (**c-3**) and (**d-3**) display result of LIWWF.

In order to perform a comprehensive analysis of the proposed methods, we selected four pairs of stereo remote sensing images obtained by UAV to the experimental analysis. Seeing in Figure 7, these UAV images contain different distortion including affine distortion, rotation, blur, and noise. The MSER+SIFT matching algorithm (abbreviated as MSER), DWT-MSER, which applies DWT to smooth images before MSER detecting, the WαSH + SIFT matching algorithm (abbreviated as WαSH), DWT-WαSH, and KAZE [24] are compared in this experiment. KAZE is a multiscale feature detection and description algorithm in nonlinear scale spaces [24]. It is similar to SIFT, but more robust than SIFT. In the MSER/WαSH method, the MSER/WαSH detector is applied to extract local features, which are consequently described by the SIFT-describing algorithm. All of the comparison methods determine matches by using NNDR. The threshold value is set to 0.8, which is equal to that of the proposed method. The matches of the proposed methods are depicted in Figure 7.

The matching results are presented in Table 2. *N*_t_ is the number of total matches; *N*_c_ is the number of correct matches which have overlap error lower than 50%; *C*_r_ is the correct matching rate that is equal to the percentage of *N*_c_ and *N*_t_.

Based on the results displayed in Table 1, the analysis is as follow:

Comparing *N*_t_ of MSER and DWT-MSER, we can find that the number of total matches decreased when DWT was paired to MSER. Nevertheless, when DWT was paired to WαSH, it increased. The reason is that MSER detected much fewer features on the smoothed images than on the original images, while WαSH detected a similar number due to edge detecting process. The values of *N*_t_ and *N*_c_ of WWF, IWWF, and LIWWF were improved relative to WαSH, which is consistent with the result in Section 5.1. This is because WWF, IWWF, and LIWWF detect WαSH features on three images, which increase the number of features. Among the WαSH-based methods (i.e., WαSH, DWT-WαSH, WWF, IWWF, and LIWWF), LIWWF achieved the largest number of total matches and correct matches, followed by IWWF. *C*_r_ values of IWWF and LIWWF were relatively stable. For images in Figure 7b, which contain simple texture and large rotation distortion, the correct matching rate of MSER, DWT-MSER, WαSH, DWT-WαSH, and WWF were less than 50% with a low *N*_t_ that is difficult to estimate the homography matrix between image pairs, while IWWF and LIWWF obtained relatively reliable matches. For images in Figure 7c containing affine distortion and slight noise, *C*_r_ values of KAZE and proposed methods were all above 90%, and the correct matching rate of WWF reached to 100%. KAZE obtained the largest value of *N*_t_ than other methods. However, KAZE obtained a 35.71% correct matching rate, which is extremely low, in Figure 7a that may cause failure in calculating the homography matrix between the image pairs. IWWF was the only method that achieved the correct matching rate no less than 50% for all of the test images. Therefore, IWWF is the most stable to different kinds of distortion among these comparative methods.

## 6. Conclusions

The WαSH local invariant feature has advantages in distinctiveness and robustness. However, image matching based on WαSH features performs a small number of matches and noise sensitivity. Focusing on addressing these problems, this paper improved WαSH based on 2D-DWT and proposed three methods of WWF, IWWF, and LIWWF for image matching. Experiments on sequences of affine distortion, scale distortion, illumination change images, and a sequence of simulated noise images showed that the WWF, IWWF, and LIWWF acquired more matches and better performance than WαSH. For remote sensing images with less affine distortion and slight noise, the correct matching rate of KAZE and proposed methods were higher than MSER and WαSH, and the correct matching rate of WWF reached to 100%. Nevertheless, for images containing severe distortion, KAZE obtained extremely low correct matching rate which is unacceptable for the homography matrix calculating, while IWWF achieved 71.42% correct matching rate. IWWF was the only method that achieved the correct matching rate no less than 50% for all of the test images and was the most stable comparing with MSER, DWT-MSER, WαSH, DWT-WαSH, KAZE, WWF, and LIWWF. 

The proposed methods are 2–3 times slower than WαSH because they detected WαSH features on three sub-images. In addition, the normalizing and resampling processes make the proposed methods moderate increase in computational cost. The proposed methods improved the number of matches and the stability at the expense of computational efficiency. We are going to focus on speeding up the proposed method in future work.

## Figures and Tables

**Table 1:** Repeatability and correct matching rate of WαSH features on different image layer of the 5th image pair of Leuven.

WαSH Features on	Repeatability	*C*_r_
Original image	53.719%	67.68% (described on original image)
LL layer	48.7395%	79% (described on original image)
		88.36% (described on LL layer)
LH layer	52.5424%	62.5% (described on LH layer)
HL layer	38.2022%	84.62% (described on HL layer)

**Table 2:** Results of matching for stereo remote sensing image pairs.

		MSER	DWT-MSER	WαSH	DWT-WαSH	KAZE	WWF	IWWF	LIWWF
Figure 7a	*N*_t_	9	1	7	10	28	15	14	18
	*N*_c_	4	0	6	6	10	7	10	10
	*C*_r_	44.44	0.00	85.71	60.00	35.71	46.67	71.43	55.56
Figure 7b	*N*_t_	34	11	13	16	206	21	22	28
	*N*_c_	7	3	6	7	111	5	11	14
	*C*_r_	20.59	27.27	46.15	43.75	53.88	23.81	50.00	50.00
Figure 7c	*N*_t_	66	55	15	34	1236	18	34	40
	*N*_c_	46	38	13	27	1114	18	33	36
	*C*_r_	69.70	69.09	88.67	79.41	90.13	100.00	97.06	90.00
Figure 7d	*N*_t_	19	9	14	30	199	28	36	42
	*N*_c_	8	4	7	12	82	13	19	20
	*C*_r_	42.11	44.44	50.00	40.00	46.23	46.43	52.78	47.62
